# Combining Magnetic Resonance Imaging with Systemic Monocyte Evaluation for the Implementation of GBM Management

**DOI:** 10.3390/ijms22073797

**Published:** 2021-04-06

**Authors:** Carolina Giordano, Giovanni Sabatino, Simona Romano, Giuseppe Maria Della Pepa, Martina Tufano, Quintino Giorgio D’Alessandris, Simone Cottonaro, Marco Gessi, Mario Balducci, Maria Fiammetta Romano, Alessandro Olivi, Simona Gaudino, Cesare Colosimo

**Affiliations:** 1 UOC Radiodiagnostica e Neuroradiologia, Istituto di Radiologia, Fondazione Policlinico Universitario “A.Gemelli” IRCCS, Università Cattolica S.Cuore, 00168 Roma, Italy; carolinagiordano91@gmail.com (C.G.); cottonaro.simone90@gmail.com (S.C.); simona.gaudino@policlinicogemelli.it (S.G.); cesare.colosimo@policlinicogemelli.it (C.C.); 2 UOC Neurochirurgia, Istituto di Neurochirurgia, Fondazione Policlinico Universitario “A.Gemelli” IRCCS, Università Cattolica S.Cuore, 00168 Roma, Italy; giovanni.sabatino@materolbia.com (G.S.); giuseppemaria.dellapepa@policlinicogemelli.it (G.M.D.P.); quintinogiorgio.dalessandris@policlinicogemelli.it (Q.G.D.); alessandro.olivi@policlinicogemelli.it (A.O.); 3 UOC of Neurochirurgia “Ospedale Mater Olbia”, 07026 Olbia, Italy; 4 Dipartimento di Medicina Molecolare e Biotecnologie Mediche, Università di Napoli Federico II, Via Pansini, 5, 80131 Napoli, Italy; simona.romano@unina.it (S.R.); martina.tufano@unina.it (M.T.); 5 UOS di Neuropatologia, UOC Anatomia Patologica, Fondazione Policlinico Universitario “A.Gemelli” IRCCS, Università Cattolica S.Cuore, 00168 Roma, Italy; marco.gessi@policlinicogemelli.it; 6 UOC di Radioterapia Oncologica, Dipartimento di Diagnostica per Immagini, Radioterapia Oncologica ed Ematologia, Fondazione Policlinico Universitario “A.Gemelli” IRCCS, Università Cattolica S.Cuore, 00168 Roma, Italy; mario.balducci@policlinicogemelli.it

**Keywords:** glioblastoma multiforme, MRI, pseudoprogression, liquid biopsy, FKBP51s

## Abstract

Magnetic resonance imaging (MRI) is the gold standard for glioblastoma (GBM) patient evaluation. Additional non-invasive diagnostic modalities are needed. GBM is heavily infiltrated with tumor-associated macrophages (TAMs) that can be found in peripheral blood. FKBP51s supports alternative-macrophage polarization. Herein, we assessed FKBP51s expression in circulating monocytes from 14 GBM patients. The M2 monocyte phenotype was investigated by qPCR and flow cytometry using antibodies against PD-L1, CD163, FKBP51s, and CD14. MRI assessed morphologic features of the tumors that were aligned to flow cytometry data. PD-L1 expression on circulating monocytes correlated with MRI tumor necrosis score. A wider expansion in circulating CD163/monocytes was measured. These monocytes resulted in a dramatic decrease in patients with an MRI diagnosis of complete but not partial surgical removal of the tumor. Importantly, in patients with residual tumor, most of the peripheral monocytes that in the preoperative stage were CD163/FKBP51s− had turned into CD163/FKBP51s+. After Stupp therapy, CD163/FKBP51s+ monocytes were almost absent in a case of pseudoprogression, while two patients with stable or true disease progression showed sustained levels in such circulating monocytes. Our work provides preliminary but meaningful and novel results that deserve to be confirmed in a larger patient cohort, in support of potential usefulness in GBM monitoring of CD163/FKBP51s/CD14 immunophenotype in adjunct to MRI.

## 1. Introduction

Glioblastoma (GBM) is the most common malignant primary brain tumor, accounting for 54% of all gliomas and 16% of all primary brain tumors [1]. Despite advances in treatment modalities, GBM remains an incurable tumor with an average survival of fewer than two years. Standard treatment consists of maximal surgical resection followed by radio-chemotherapy according to Stupp protocol [2]. This protocol of treatment consists of radiotherapy plus continuous daily temozolomide (TMZ), from the first to the last day of radiotherapy, followed by six cycles of adjuvant TMZ, each 28-day cycle [2]. Surgical resection is a critical step because the invasive tumor cells that remain after surgery and survive the treatments are likely responsible for tumor recurrence [3]. Magnetic resonance imaging (MRI) remains the reference standard for the pre-operative, post-operative, and follow-up evaluation of glioma patients [4]. In the pre-surgical assessment of tumors, MRI provides information for surgical approaches and/or for radiation treatment planning. Post-operative and follow-up MRI assess the extent of surgical resection and treatment response. GBM is known to have a strong tendency to relapse after surgery and radio/chemotherapy treatments. At the time of relapse, the median survival is only 5–7 months despite therapy [5]. Among the most common reasons for non-radical resection is the inability to adequately differentiate the tumor from the normal brain parenchyma during surgery [6] because of homogenous texture and color under white light, along with infiltration into the surrounding brain. Selective immunofluorescence techniques, based on 5-aminolevulinic acid and the ultraviolet light microscope to guide supramarginal resection, do not completely exclude a certain tumor infiltration in non-fluorescent samples [7].

Morphological MRI often fails to identify and quantify the presence of infiltration in GBM peritumoral non-enhanced areas, resulting in inaccuracy in the assessment of GBM-invasive margin. Moreover, morphological MRI fails to distinguish the marked enhancement in the tumor bed caused by radionecrosis, without actual tumor, from true tumor progression. Pseudoprogression is a real challenge for the neuroradiologist; on MRI, it can mimic a tumor progression, showing an increase in contrast-enhancing and lesion size [8,9]. Subsequent clinical improvement or stabilization without any further treatment will confirm the diagnosis of pseudoprogression [8,9]. Even if non-morphological imaging [perfusion-weighted imaging (PWI), diffusion-weighted imaging (DWI), diffusion-tensor imaging (DTI), magnetic resonance spectroscopy (MRS)] and/or positron-emission tomography (PET)-MRI are suggested as useful tools to recognize tumor progression [9], the introduction of new non-invasive diagnostic modalities, as a tumor-related biomarker, could help reveal the invisible tumor, in adjunct to MRI.

GBM is heavily infiltrated with monocytic cells, namely, tumor-associated macrophages (TAMs) with features of immunosuppressive myeloid-derived suppressor cells [6]. TAMs originate from two independent sources: brain-resident microglia and bone marrow-derived monocytes [10,11]. Because of their high plasticity, the vast majority of GBM-infiltrating monocytes are in situ-educated by the tumor [12,13,14]. Friebel and coworkers identified CD163 as a general marker of tumor-invading monocyte-derived macrophages [12]. CD163 represents the most classical and most specific marker of TAMs and is also a prognostic factor in glioma and other types of cancer [15]. The programmed death ligand-1 (PD-L1/B7-H1) is a further important TAM marker that can inhibit the activation of T lymphocytes and enhance the immune tolerance of tumor cells, thereby achieving tumor immune escape. [16]. Zhu and coworkers [17] analyzed gene expression data and clinical information of about 1000 glioma patients from The Cancer Genome Atlas (TCGA) and Chinese Glioma Genome Atlas (CGGA) databases, finding a positive correlation of tumor PD-L1 expression with M2 polarization of infiltrating macrophages (M2-TAMs) along with an unfavorable prognostic significance of PD-L1 expression on GBM.

In addition to the intratumoral increase of TAMs, myeloid-derived suppressor cells can be found numerous in the blood of advanced GBM patients [10,13,14]. Bloch and coworkers found that circulating monocytes in GBM patients had significantly increased expression of PD-L1 compared with healthy controls [18]. Intratumoral and systemic tumor-associated monocytes play a relevant role in promoting immunosuppression [10,18], supporting neoplastic progression [3] and contrasting anti-PD1 therapy [10,19]. 

We have previously found that, in melanoma patients, resistance to anti-PD1 is associated with an expansion of a peripheral blood mononuclear cell (PBMC) subset marked by CD14/PD-L1/FKBP51s [20,21] and expressing increased transcript levels of ARG1 and MSR-1 [21]. Moreover, we demonstrated that FKBP51s plays a role in the alternative-polarization of monocytes (M2) and showed that FKBP51s-forced expression in macrophage reduced the ability of this accessory cell to co-stimulate lymphocyte proliferation [21]. FKBP51 is a peptidyl-prolyl cis-trans isomerase (PPIase) encoded by *FKBP5* gene, which is endowed with a protein–protein interaction (tetratricopeptide or TPR) domain at C-terminus [22]. Thanks to its structure, FKBP51 serves either as a scaffold and isomerase that organizes protein complexes, assuring an optimal fit and function [22]. First identified as a component of the steroid-receptor complex [23], FKBP51 was found to interact with IKK kinase complex subunits, thus contributing to their assembly and maximizing catalytic function on the IκB substrate [24]. FKBP51 regulates the basal and inducible activation of NF-κB [24]. FKBP51 is physiologically expressed by immune system cells [25], where it virtually exerts a relevant function given the pivotal role of NF-κB in shaping the immune system and sustaining the innate and adaptive immune response. The spliced isoform of this immunophilin, i.e., FKBP51s, was for the first time identified as generated by immune-inhibitory signals emanating from PD-L1/PD1 interaction [26]. This isoform retains the PPIase activity but lacks the TPR domain and has a distinct C-terminus. It acts as a PD-L1 co-chaperone, with a role in PD-L1 protein maturation [27].

The present work aimed to assess the suitability of FKBP51s for identification of a monocyte subset in peripheral blood of GBM patients that could provide supportive information to MRI for patient evaluation. To this end, we performed immunostaining of peripheral blood monocytes with two recognized M2 surface markers, namely, PD-L1 and CD163, along with FKBP51s. We found that PD-L1 expression on monocytes was strictly related to tumor necrosis, which is a hallmark feature of GBM tumors [28]. CD163+ monocytes were highly represented in peripheral blood of all patients, regardless of tumor necrosis. CD163+ monocytes partly co-expressed FKBP51s. The complete tumor removal was accompanied by a significant decrease in CD163+ cell subset. In patients who had an MRI diagnosis of residual tumor, most of the peripheral monocytes that in the preoperative stage were CD163+ FKBP51s− had turned into CD163+ FKBP51s+.

## 2. Results

### 2.1. Increased M2 Gene Expression in PBMCs of GBM Patients

**Figure 1:**
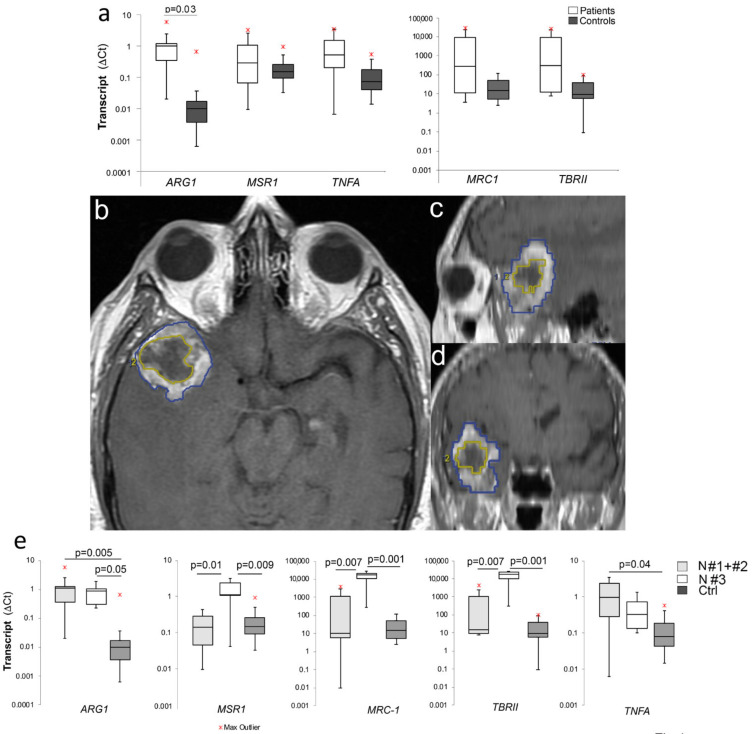
M2 gene expression profile in peripheral blood of glioblastoma (GBM) patients (**a**) Box plots of ARG1, MSR1, MRC1, TBR II, and tumor necrosis factor-α (TNFA) transcript levels in RNA extracted by 11 patients and 9 healthy donors’ peripheral blood mononuclear cells (PBMCs). (**b**–**d**) T1 TSE post-gadolinium images, axial section (**b**), and sagittal (**c**) and coronal (**d**) plane reconstructions: Region of interest (ROI) 1 (blue) indicate the tumor volume, ROI 2 (yellow) the necrosis volume. (**e**) MSR1, MRC1, TBR II, and TNFA transcript levels according to the necrosis score (N#3 = 4, N#1 + #2 = 8). Graphs were generated using Microsoft Excel.app 16.43.

M2 macrophages display shifted arginine metabolism towards the production of ornithine and polyamines via arginase. Besides arginase (ARG1), additional M2 markers are the macrophage scavenger receptor MSR1, the mannose receptor C type 1 MRC1, and the transforming growth factor-β-receptor II TBRII [29]. To address whether PBMCs of GBM patients contain an expansion of M2 monocytes, which can be used for diagnostic purposes, we measured the levels of ARG1, MSR1, MRC1, and TBRII by qPCR. The transcript level of the pro-inflammatory cytokine tumor necrosis factor-α (TNFA), which is instead associated with an M1 profile [29], was also measured. Apparently, patients, in comparison with control donors, had increased only ARG1 transcript levels in their PBMCs (Figure 1a). Because tumor necrosis profoundly affects the immunosuppressive microenvironment [3], we analyzed such gene expression in relation to tumor necrosis level, as assessed by MRI [30]. Representative MRI of tumor necrosis is shown in Figure 1b–d. Among the 12 patients analyzed, 5 patients had a necrosis score (NS) #3. The remaining seven patients included 3 NS#1 and 4 NS#2. NS#3 patients showed increased MSR1, MRC1, and TBRII in comparison with NS < #3 patients and controls (Figure 1e). Interestingly, the levels of TNFA in NS < #3, but NS#3, were higher than in controls (Figure 1e). These results suggest the presence of M2 monocytes in peripheral blood of GBM patients, which is positively influenced by tumor necrosis.

### 2.2. High Frequency of PD-L1+ and CD163+ Monocytes in Peripheral Blood of GBM Patients

**Figure 2:**
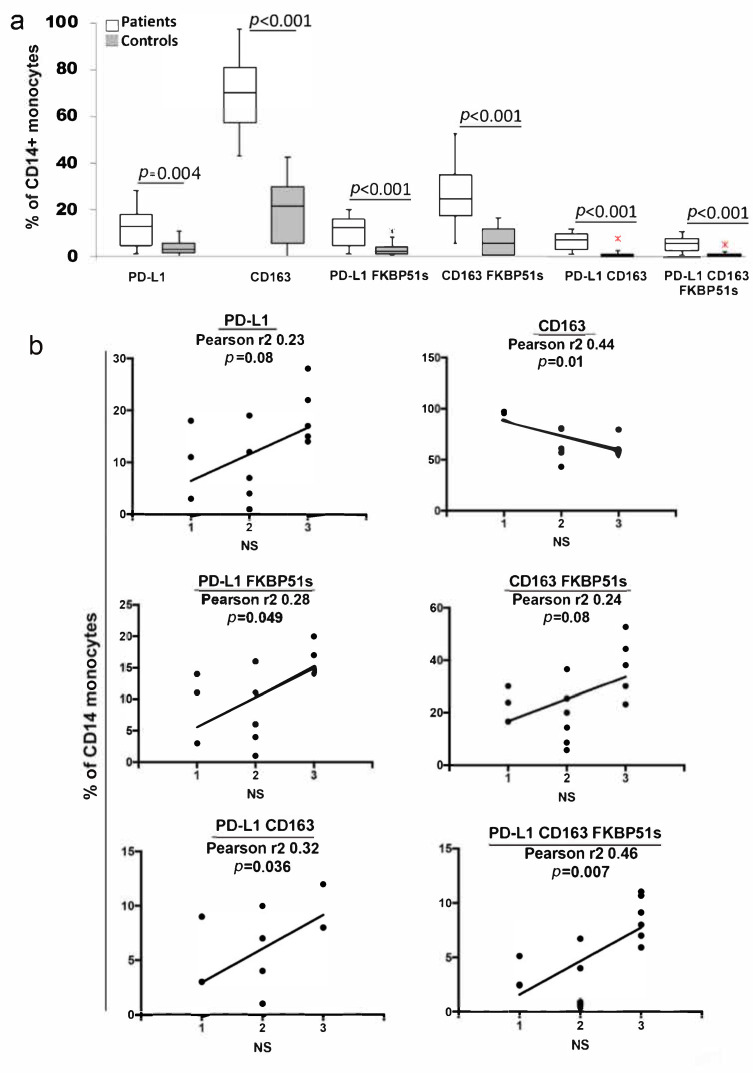
PD-L1+ peripheral monocytes in GBM patients (**a**) Box plots of counts of the whole PD-L1+ and CD163+ monocytes and their subsets co-expressing FKBP51s+ or a combination of PD-L1 and CD163 in the study population (14 patients and 14 healthy donors). Counts were performed on CD14-gated PBMCs. The graph was generated using Microsoft Excel.app 16.43. (**b**) Linear regression with Pearson correlation of M2 monocyte subsets and necrosis score. Graphs were generated using Prism GraphPad 7.0a Macintosh. NS = necrosis score.

Previous studies draw attention to PD-L1 [18] and CD163 [12] as TAM markers. FKBP51s is a spliced immunophilin expressed by M2 monocytes [21]. In an attempt to characterize the phenotype of systemic M2 monocytes of GBM patients, we performed a quadruple staining (CD14, CD163, PD-L1, FKBP51s) of PBMCs from 14 GBM patients and 14 control donors. Representative flow cytometry histograms of CD14/SSc-gated monocytes and the CD14+ subsets analyzed are shown in Figure S1. In GBM patients, we found that PD-L1+ monocytes (12.2% + 8.2) were higher than in control subjects (3.9% + 3.4 (*p* = 0.0037) (Figure 2a). CD163 stained 71.01% + 16.9 of peripheral monocytes of GBM patients while control values were 18.9% + 13.4 (*p* < 0.0001) (Figure 2a). Both PD-L1 and CD163 subsets included a fraction of FKBP51s+ cells. More precisely, PD-L1/FKBP51s+ and CD163/FKBP51s+ were, respectively, 10.9% + 6.7 and 26.4% + 13.3 in patients, and 3.0% + 2.8 (*p* < 0.001) and 6.6% + 6.0 (*p* < 0.001) in controls (Figure 2a). A small subset co-expressed PD-L1 and CD163: values in patients and controls were 6.5% + 4.2 and 1.3% + 2.0, respectively, *p* = 0.002 (Figure 2a). PD-L1/CD163/FKBP51s+ monocytes were also measured (5.3% + 3.4 and 0.9% + 1.3, for patients and controls, respectively; *p* < 0.001) (Figure 2a). These findings suggest an expansion of distinct subsets of monocytes expressing M2 markers in PBMCs of GBM patients. Tumor necrosis represents an important prognostic factor in GBM [28,31]. To address whether these monocyte subsets were influenced by the tumor, we measured their counts in relation to the previous classification of patients according to MRI necrosis score. The 14 patients comprised three NS#1, five NS#2, and six NS#3. As shown in Figure 2b, the PD-L1 subsets co-expressing FKBP51s or CD163 (alone or in combination) but whole PD-L1 appeared to positively correlate with NS. Our result is in agreement with findings by Bloch et al. [18] who found a PD-L1/CD163+ monocyte subset associated with worst prognosis in GBM. A negative correlation with NS was calculated for CD163+ monocytes (Figure 2b). Co-expression of FKBP51s appeared to revert the trend of CD163 monocytes (Figure 2b). Correlative analysis between the monocyte subsets and other tumor information obtained with T1, T2, T2 FLAIR (fluid attenuated inversion recovery), susceptibility-weighted imaging (SWI), and DWI sequences are shown in Figures S2–S7. Tumor features as ependymal enhancement (Figure S2), corpus callosum infiltration (Figure S3), midline shift (Figure S4), macroscopic hemorrhage (Figure S5), the proportion of edema (Figure S6), and intratumoral susceptibility signal intensity (ITSS) (Figure S7) did not appear to affect the peripheral monocyte phenotype.

### 2.3. CD163+FKBP51s+ Monocytes Are Sensitive to Tumor Removal

**Figure 3:**
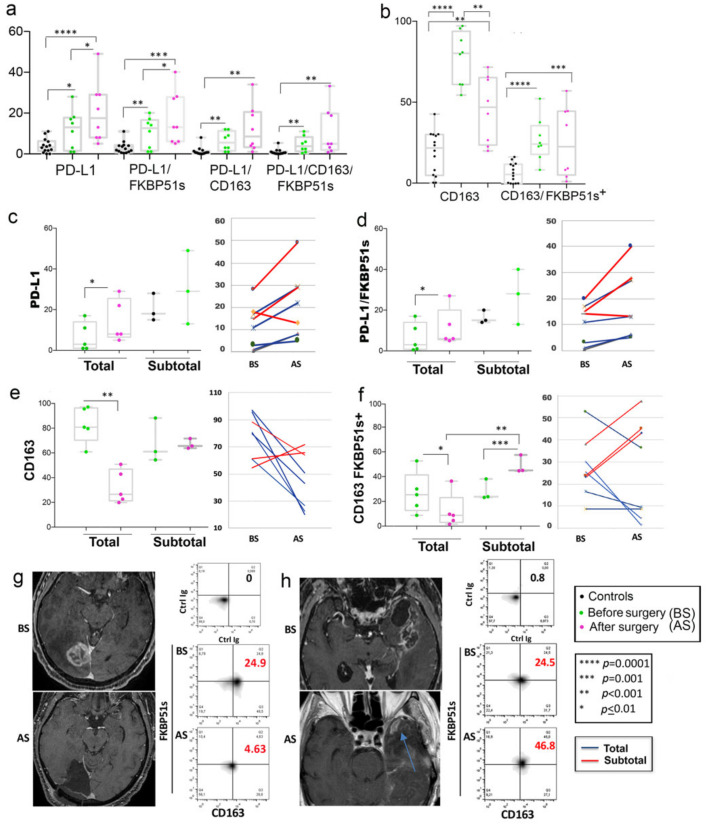
Effect of tumor removal on M2 immunophenotype (**a**) Boxplots of pre-operative and post-operative counts of whole PD-L1+ monocytes and their subgroups. (**b**) Boxplots of pre-operative and post-operative counts of whole CD163+ monocytes or CD163/FKBP51s+ monocytes. (**c**–**f**) Boxplots of pre-operative and post-operative counts are shown according to total or subtotal tumor removal: whole PD-L1+ monocytes (**c**), PD-L1/FKBP51s+ monocytes (**d**), whole 163+ monocytes (**e**), CD163/FKBP51s monocytes (**f**). In any panel, the line graph on the right shows the post-operative changes for each patient. Boxplots were generated using Prism GraphPad 7.0a Macintosh; line graphs were generated using Microsoft Excel.app 16.43. (**g**) T1 W post-gadolinium images of a patient with total resection and (**f**) a case of subtotal resection (**h**). Blue arrow indicates the residual part of the tumor. Before-surgery images (BS) and after-surgery images (AS) are shown along with flow cytometry histograms of CD163/FKBP51s+ monocytes (highlighted in red).

We investigated the effect of tumor removal on the counts of the previously identified monocyte subsets. Due to the reduction in cooperative behavior, only 8/14 patients received combined blood analysis and MRI, approximately 1 month after surgery and before radio-chemotherapy according to Stupp [2]. As for post-operative MRI tumor assessment [7], five patients (two NS #1 + two NS #2 + one NS #3) received total or near-total (98–100%) resection, while three patients (one NS #1 + two NS #3) received a subtotal (89%) or less than subtotal (78% and 67%) resection. In comparison with pre-surgery values, whole PD-L1+ and PD-L1/FKBP51s+ monocytes increased after surgery (Figure 3a). Differently, a significant decrease of whole CD163+ monocytes, but CD163/FKBP51s+, was measured after surgery (Figure 3b). Interestingly, totally resected patients showed an increase in PD-L1+ monocytes, including PD-L1/FKBP51s+ (Figure 3c,d), but a decrease in CD163 monocytes, including CD163/FKBP51s+ (Figure 3e,f). In patients with incomplete resection, no significant changes in whole CD163 monocyte count was registered after surgery, but the fraction co-expressing FKBP51s+ resulted in significant increase (Figure 3e,f). Figure 3 also shows representative MRI and correspondent CD163/FKBP51s+ monocytes from total (Figure 3g) or subtotal (Figure 3h) resected patients.

**Figure 4:**
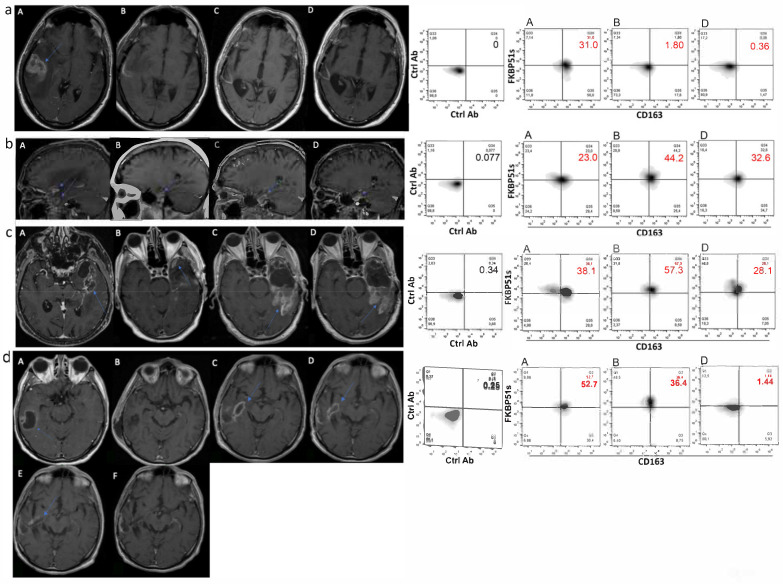
MRI and CD163/FKBP51s+ monocytes on follow-up. Axial T1 images after contrast medium administration: the glioblastoma before surgery in A, the immediate post-operative image in B, post-radio-chemotherapy in C, and post-adjuvant temozolomide (TMZ) in D. On the right, correspondent flow cytometry of CD163/FKBP51s+ monocytes. (**a**) The glioblastoma before surgery in the right temporal lobe. The immediate post-operative image shows a macroscopically complete exeresis of the tumor. Images C and D show no disease progression. (**b**) The glioblastoma before surgery in the right temporal lobe. The immediate post-operative image shows a residual tumor. Images C and D show the stability of the residual tumor. (**c**) The glioblastoma before surgery in the left temporal lobe. The immediate post-operative image shows a residual tumor. Image C highlights a disease progression, which tends to be stable in subsequent D control. (**d**) The glioblastoma before surgery in the right temporal lobe. The post-operative image shows a macroscopically complete exeresis of the tumor. C and D images show a thick impregnation area along the deepest portion of the surgical cave suspected for disease progression, which tended to regress in subsequent controls (E, F).

Association of CD163+FKBP51s+ monocytes with residual tumor was also suggested by the analysis of immunophenotype in concert with MRI in four patients (two cases of complete and two of incomplete resection) that received immunophenotype after Stupp therapy. Figure 4 shows MRI images in sequence of the patients: A, before surgery; B, the immediate post-operative; C, post-radio-chemotherapy; D, post-adjuvant TMZ. In comparison with preoperative values, the patient in Figure 4a showed a reduced level of CD163/FKBP51s+ monocytes after surgery and at the follow-up along with MRI diagnosis of total tumor resection (B) and no disease progression (C, D). Patients in Figure 4b,c showed an increased level of CD163/FKBP51s+ monocytes after surgery along with MRI diagnosis of subtotal tumor resection (B). Although slightly reduced by Stupp therapy, the level of such monocytes remained still high at follow-up (D). In Figure 4d, a case of pseudoprogression is shown. After an apparent radical surgery (B), on follow-up, MRI revealed a thick impregnation area along the deepest portion of the surgical cave, suggesting tumor recurrence (C, D). CD163/FKBP51s+ monocytes decreased after surgery and almost disappeared during adjuvant TMZ, supporting the possibility that contrast agent enhancement of the lesion did not correspond to true tumor growth. MRI at 4 and 7 months (E and F, respectively) after the end of adjuvant TMZ showed the progressive regression of the lesion.

## 3. Discussion

**Figure 5:**
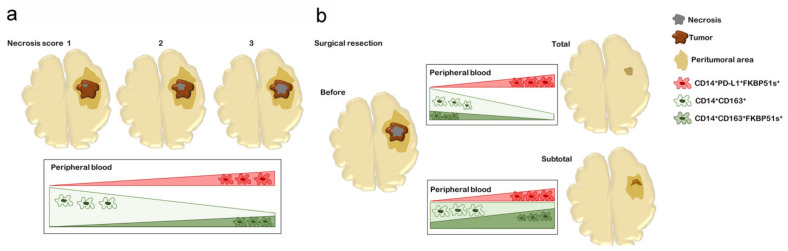
Visual summary of the main findings of the article. The cartoon shows that FKBP51s marked CD163+ monocytes (green) or PD-L1+ monocytes (red) that responded in a different way to tumor necrosis score (**a**) or extent of surgical resection (**b)**. (**a**) PD-L1+FKBP51s+ monocytes increased with the tumor necrosis score. By contrast, CD163+ monocytes decreased with the necrosis score. This trend was reversed, albeit not significantly, in CD163+FKBP51s+ monocytes. (**b**) The PD-L1+FKBP51s+ subset had a postoperative increasing tendency, both in the cases of total and subtotal tumor removal. The CD163^+^FKBP51s+ monocytes decreased upon total removal, while they increased after subtotal removal.

Monocytes are highly plastic cells that undergo specific differentiation depending on the local tissue environment. They respond to environmental cues within tissues to differentiate into distinct functional phenotypes. We have previously shown that the splicing of FKBP51 is an event that concurs with the acquisition of M2 features, namely, IL-10, STAT-3, and PD-L1 upregulation [21]. Chen and coauthors demonstrated that the transformation of bone-marrow-derived macrophages in TAMs occurs very early during GBM development due to direct interaction with neoplastic cells in the perivascular niche [11]. Their tumor-promoting effects operate from the initial stage of GBM development [11]. IL-10 produced by glioma cells has also been found to play a role as an initial chemotactic agent to recruit monocytes and a driver of immunosuppressive loops between cancer cells and macrophages within the tumor microenvironment [32,33]. IL-10 cooperates with other not yet identified tumor-derived soluble factors to promote TAM development [18]. Although the dynamics of the cross-talk between glioma cells and TAMs remain scarcely known, it is widely accepted that TAMs are a heterogeneous population that contributes significantly to the creation and maintenance of immunosuppression and tumor progression [14]. There is still a lack of knowledge regarding the differential composition and functions of TAMs [14]. However, also in contexts different from cancer, it is now clear that the M2 macrophages encompass a functionally diverse group of macrophages [34,35]. In our study, ARG1 levels were increased in all patients, as previously described [36]; differently, MSR1, MRC1, and TBRII expressions were remarkable only in patients with high necrosis score. These results support the concept of M2 macrophage heterogeneity. Consistently, using quadruple staining, we identified several monocyte subsets. Some phenotypes that were co-expressing PD-L1, FKBP51s, and/or CD163 appeared to be correlated with the necrosis score, suggesting that changes in the tumor microenvironment of GBM could influence their development. We found that CD163 is the most represented M2 marker of GBM peripheral monocytes. Unexpectedly, it was inversely correlated with the necrosis score. Gabrusiewicz et al. found that glioblastoma-derived exosomes induce PD-L1 expression on human monocytes [37]. It could be hypothesized that, in circumstances such as necrosis, stimuli from the microenvironment preferentially upregulate subsets of PD-L1+ monocytes. CD163 monocytes appeared to respond to the surgical removal of the tumor. In subtotal resected patients, most of the peripheral CD163 monocytes that were FKBP51s− in the preoperative stage had turned into FKBP51s+, suggesting that the persisting tumor stimulated the development of this monocyte subset. Figure 5 illustrates how PD-L1 and CD163 monocyte subsets change according to necrosis score and surgical resection. After Stupp therapy, while totally resected patients showed CD163/FKBP51s+ monocytes almost disappeared, patients with MRI diagnosis of persistent tumor had sustained levels in such circulating monocytes. This observation opens the possibility to use CD163/FKBP51s+ monocytes to discriminate between pseudoprogression (radionecrosis) and true tumor progression.

In conclusion, in this study, MRI guided us in characterizing subpopulations of circulating monocytes associated with GBM and their possible diagnostic and prognostic relevance. The different outcomes of circulating PD-L1+ and CD163+ monocytes that respectively increased or decreased after surgery support the hypothesis that different stimuli promote their development. Whether the post-surgery increase of PD-L1 monocytes reflected a pro-healing function of this M2 monocyte subset remains to be investigated [38,39]. Even if a note of caution must be given due to a low number of patients enrolled, our study uncovered a peripheral monocyte subset useful for post-surgical assessment of GBM. It should be emphasized that the present study is hypothesis-generating and that future studies with a larger cohort of patients are needed to address the suitability of CD163/FKBP51s+ monocyte phenotype as a powerful diagnostic tool in adjunct to MRI for the management of GBM patients.

## 4. Materials and Methods

### 4.1. Patients

**Figure 6:**
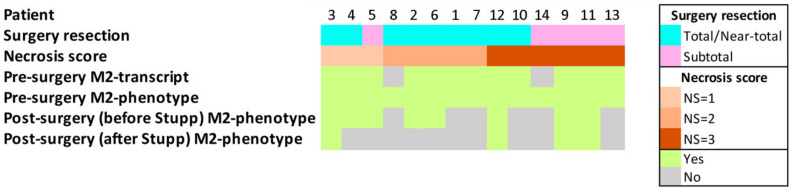
Workflow of the studied GBM patients.

The ethics committee of Fondazione Policlinico Universitario A.Gemelli approved the protocol of this prospective study (Prot 13891/18, 18/05/2018). The study was conducted in accordance with the ethical principles of the Declaration of Helsinki. Informed consent was obtained from all participants (patients and donors). From March 2019 to March 2020, we enrolled 16 consecutive patients in whom a glioblastoma was suspected on MRI. Of these 2 patients, histology ruled out glioblastoma. Therefore, 14 patients (11 males, 3 females; age, 58 ± 17 years) were included in the study (Table 1). Figure 6 illustrates the patient workflow in color.

Patients were managed according to published guidelines [40]. Patients received no corticosteroids before MRI and blood collection. Clinical information and the results of the study were handled by authorized personnel only. In compliance with patients’ rights, patient identity was kept confidential.

### 4.2. Neuropathology

All cases were classified as glioblastoma (grade IV) according to the WHO classification of tumor of the central nervous system 2016 [41]. The IDH status was evaluated using immunohistochemistry with specific IDH1 (R132H) antibody or, if required, Sanger sequencing (in particular for patients of <54 years of age).

### 4.3. Imaging and Image Analysis

All patients were scanned on an Optima MR450W (GE Healthcare, Milwaukee, WI, USA) or Philips Ingenia 1.5 T clinical scanner using an 8-channel head coil. We collected conventional anatomic MRI sequences such as T1-weighted, T1-postgadolinium (T1C), T2-weighted, T2, T2 FLAIR, susceptibility-weighted imaging (SWI), as well as diffusion-weighted (DWI) and Dynamic Susceptibility Contrast (DSC) sequences. DSC series were processed using Olea Sphere software. Specific acquisition parameters are given (Tables S1 and S2). Pre-operative MRIs were analyzed in consensus, blinded to the clinical data; by a neuroradiologist with over 10 years of experience (SG); and by a radiologist resident with 4 years of experience (CG). Qualitative analysis was based on the following morphological features: tumor location, macroscopic hemorrhage, ependymal enhancement, corpus callosum infiltration, and midline shift (Table 1). Quantitative analysis includes measurement of tumor volumes, the score of necrosis, intratumoral susceptibility signal intensity (ITSS), and edema (Table 1). The volumes of both the tumor and the zone of necrosis were calculated with a dedicated workstation (Advantage Windows, version 4.6, GE Healthcare, Milwaukee, WI, USA) using an analysis based on freehand-drawn Region of Interests (ROIs) with the semiautomatic system. Tumor volumes were contoured on T1-weighted contrast-enhanced images. The volume of necrosis was contoured by the parts of the tumor that were not enhanced. The relative extent of necrosis within the tumor was obtained by a simple division of their volumes (necrosis/tumor ratio = N/T) [30]. A necrosis score was assigned on the basis of the percentage of intratumoral necrosis (score 1: <5%, score 2: between 5% and 20%, score 3: more than 20%) [30]. ITSS was valued on SWI images and scored according to previous literature as follows: 0 = absence of ITSS, 1 = presence of 1–10 ITSS, 2 = presence of ≥11 ITSS [42]. Perilesional edema was detected on T2 weighted images and scored as 0: less than half of the entire abnormality is estimated to represent vasogenic edema, or 1: more than half of the entire abnormality is estimated to represent vasogenic edema [43]. Post-operative MRI (acquired within 1 month from surgery) was analyzed in consensus by S.G. and C.G. for the evaluation of the presence of a residual tumor, and where it was present, the volume was calculated (as reported above for the pre-operatory tumor volume). According to Della Pepa [7], the postoperative stratification of patients was total or supramarginal resection (≥100%); near-total resection (90–99%); subtotal resection (80–89%); less than subtotal resection (<80%).

### 4.4. Peripheral Blood Mononuclear Cell (PBMC) Isolation

PBMCs were isolated from the blood of 14 patients with glioblastoma multiforme (GBM) and 14 age- and sex-matched healthy donors. Five milliliters of blood was collected in a sterile K3EDTA vacutainer tube. PBMCs were separated by differential centrifugation through a Ficoll–Hypaque density gradient (Histopaque-1077, Sigma-Aldrich, St. Louis, MO, USA), washed, and resuspended in 5% fetal bovine serum (FBS)/Roswell Park Memorial Institute (RPMI) 1640 medium (Biowest, Nuaillè, France). After the count, PBMCs were processed for analysis by immunofluorescence (see flow cytometry analysis paragraph for further details).

### 4.5. Flow Cytometry Analysis

BD-Pharmigen Fc block (2.5 μg/106 cells) was used to minimize non-specific binding of immunoglobulins to Fc receptors, prior to the immuno-staining [21]. PBMCs were counted [44] and resuspended at the concentration of 2 × 106/mL. For this purpose, 5–10 μL (in accordance with concentration and manufacturer’s instruction) of mouse monoclonal antibody recognizing the typical cluster differentiation (CD) was added to 50 μL of PBMC suspension in order to perform a multiple immunofluorescence stainings, as previously described [21]. Cells were incubated for 15 min in the dark at room temperature (20–25 °C). The following antibodies were used: anti-CD14-PerCP (TÜK4 clone; Miltenyi Biotec, Bergisch Gladbach, Germany), anti-PD-L1-phycoerythrin (PE) (MIH1 clone; eBioscience, Thermo Fisher Scientific, Waltham, MA, USA), anti-CD163-APC (GHI/61; Molecular Probes, Thermo Fisher Scientific; Waltham, MA, USA). Next, 200 μL of a fixation/permeabilization buffer (BD-Pharmingen Cytofix/Cytoperm Kit, San Jose, CA, USA) was added to each tube and incubated for 20 min in the dark at 4 °C. After fixation and permeabilization, the cells were further incubated for intracytoplasmic staining by direct immunofluorescence with anti-FKBP51s using anti-FKBP51s antibody conjugated with the 5-carboxyfluorescein (FAM) as previously described [26]. For each staining, a relative Ig isotype-conjugated antibody was used as a control of non-specific binding. Monocyte gating and subset counts were performed as previously described [21]. Samples were analyzed using a BD AccuriTM C6 Cytometer (Becton, Dickinson and Company BD; Bergen County, NJ, USA). The flow cytometry data were analyzed by using the FlowJo software or the C6 Accurì software.

### 4.6. qPCR

Total RNA extraction was performed using TRIzol (Sigma-Aldrich), according to the manufacturer’s instructions. One microgram of each RNA was used for complementary DNA (cDNA) synthesis with iScriptTM Reverse Transcription (Bio-Rad, Hercules, CA, USA). Gene expression was quantified by quantitative (q)PCR using SsoAdvancedTM SYBR Green Supermix (Bio-Rad) and specific qPCR primers for the relative quantitation of the transcripts, performed using co-amplified beta-actin [24] as an internal control for normalization. Absolute quantification of transcripts was calculated with the 2^-DeltaCt method (DeltaCt = Ct of the target gene − Ct of the housekeeping gene) to represent the expression differences between different patients. Oligo sequences are reported: h-ARG1-Fw: 5′-GGCTGGTCTGCTTGAGAAAC-3′; h-ARG1-Rev: 5′-CTTTTCCCACAGACCTTGGA-3′; h-MSR1-Fw: 5′-CCTCGTGTTTGCAGTTCTCA-3′; h-MSR1-Rev: 5′-CCATGTTGCTCATGTGTTCC-3′; h-MRC1-Fw: 5′-AACGGACTGGGTTGCTATCA-3′; h-MRC1-Rev: 5′- CCCGATCCCTTGTAGAGCAT-3′; h-TGFBR2-Fw: 5′-AAGGAAGGGACCCATGACAG-3′; h-TGFBR2-Rev: 5′-ATGGCCAGAAGAGAAGTGCT-3′; h-TNF-α-Fw: 5′-AGCCCATGTTGTAGCAAACC-3′; h-TNF-α-Rev: 5′-TGAGGTACAGGCCCTCTGAT-3′. Transcript levels of FKBP51s were measured as described [26].

### 4.7. Statistical Analysis

The Student’s *t*-test and Dunnett’s multiple comparisons test were used to analyze the differences between the values from two groups and multiple groups, respectively. Linear regression analysis was performed using Prism GraphPad 7.0a for Macintosh. *p* value ≤ 0.05 was considered statistically significant.

## Figures and Tables

**Table 1:** Characteristics of 14 WHO-IV grade glioma patients.

Patient	Gender	Age	Localization	Pathology	SR	NS	EE	CCI	MS	MH	ES	ITSS
1	M	61	occipital	GBM IDH wt	Total	2	NO	NO	NO	NO	1	3
2	F	45	frontal	GBM IDH mutant	Total	2	NO	NO	NO	NO	0	3
3	M	75	temporo-occipital	GBM IDH wt	Near-total	1	YES	YES	YES	YES	0	3
4	F	59	frontal	GBM IDH wt	Subtotal	1	YES	YES	YES	NO	1	3
5	M	55	temporal	GBM IDH wt	Total	1	NO	NO	YES	NO	1	3
6	M	41	temporal	GBM IDH wt	Near-total	2	YES	NO	YES	NO	0	3
7	M	63	temporal	GBM IDH wt	Total	2	NO	NO	NO	NO	0	2
8	F	60	parieto-occipital	GBM IDH wt	Near-total	2	NO	YES	YES	NO	0	3
9	M	78	temporal	GBM IDH wt	Subtotal	3	NO	NO	NO	NO	0	2
10	M	66	temporo-parietal	GBM IDH wt	Near-total	3	NO	NO	NO	NO	1	2
11	M	52	temporal	GBM IDH wt	Subtotal	3	NO	NO	NO	NO	1	3
12	M	55	temporal	GBM IDH wt	Total	3	NO	NO	NO	NO	0	3
13	M	66	parieto-occipital	GBM IDH wt	Subtotal	3	NO	YES	NO	NO	0	3
14	M	52	frontal	GBM IDH wt	Subtotal	3	NO	YES	NO	NO	0	3

SR = surgery resection; NS = necrosis score; EE = ependymal enhancement; CCI = corpus callosum infiltration; MS = midline shift; MH = macroscopic hemorrhage; ES = edema score; ITSS = intratumoral susceptibility signal intensity (ITSS) score.

## Data Availability

The authors confirm that the data supporting the findings of this study are available within the article and its Supplementary Materials.

## Supplementary Materials

The following are available online at https://www.mdpi.com/article/10.3390/ijms22073797/s1. Figure S1: Representative flow cytometry histograms of CD14/SSc-gated monocytes (left) and the CD14+ subsets analyzed. Figure S2: M2 monocyte subset representation according to ependymal enhancement. Figure S3: M2 monocyte subset representation according to corpus callosum infiltration. Figure S4: M2 monocyte subset representation according to midline shift. Figure S5: M2 monocyte subset representation according to macroscopic hemorrhage. Figure S6: M2 monocyte subset representation according to edema score. Figure S7: M2 monocyte subset representation according to intratumoral susceptibility signal intensity (ITSS). Table S1: GE MR imaging protocol. Table S2: Philips MR imaging protocol.
