# Supplementary material for: Combining Magnetic Resonance Imaging with Systemic Monocyte Evaluation for the Implementation of GBM Management
Source: Int J Mol Sci. 2021 Apr 6;22(7):3797. doi: 10.3390/ijms22073797 (PMC8038816; doi:10.3390/ijms22073797)
Supplement: Supplementary file 1 [file ijms-22-03797-s001.pdf]

Table S1

GE MR imaging protocol					
	Plane	TR (ms)	TE (ms)	FOV (mm)	Slice thickness (mm)
T2 FSE	sag	3997.0	91.0	24.0	3
T2 FSE	ax	3214.0	91.0	24.0	4
T2 FSE	cor	3615.0	91.0	24.0	4
DWI b1000	ax	10265.0	minimum	24.0	4
3D SWAN	ax	minimum	40.6	25.0	1.5 (isotropic)
T1 FSE	ax	512.0	Min Full	24.0	4
FLAIR	ax	9745.0	133.1	24.0	4
DSC Perfusion	ax	1500.0	35.0	24.0	4
T1 fs FSPGR (+C)	ax		out of Phace	25.6	1.2(isotropic)
T1 FSE (+C)	ax	590.0	Min Full	24.0	4

Table S2

Philips MR imaging protocol					
	Plane	TR (ms)	TE (ms)	FOV (mm)	Slice thickness (mm)
T2 TSE	sag	3000-5500	100	24.0	3
T2 TSE	ax	3000-5500	100	24.0	3
T2 TSE	cor	3000-5500	100	24.0	3
DWI b1000	ax	4927	90	24.0	4
3D SWI	ax	51	12	24.0	1.5(isotropic)
T1 TSE	ax	400-680	12	24.0	4
FLAIR	ax	10000	140	24.0	4
DSC Perfusion	ax	1677	40	24.0	4
T1 fs WATS (+C)	ax	13	6	24.0	1(isotropic)
T1 TSE (+C)	ax	400-680	12	24.0	4

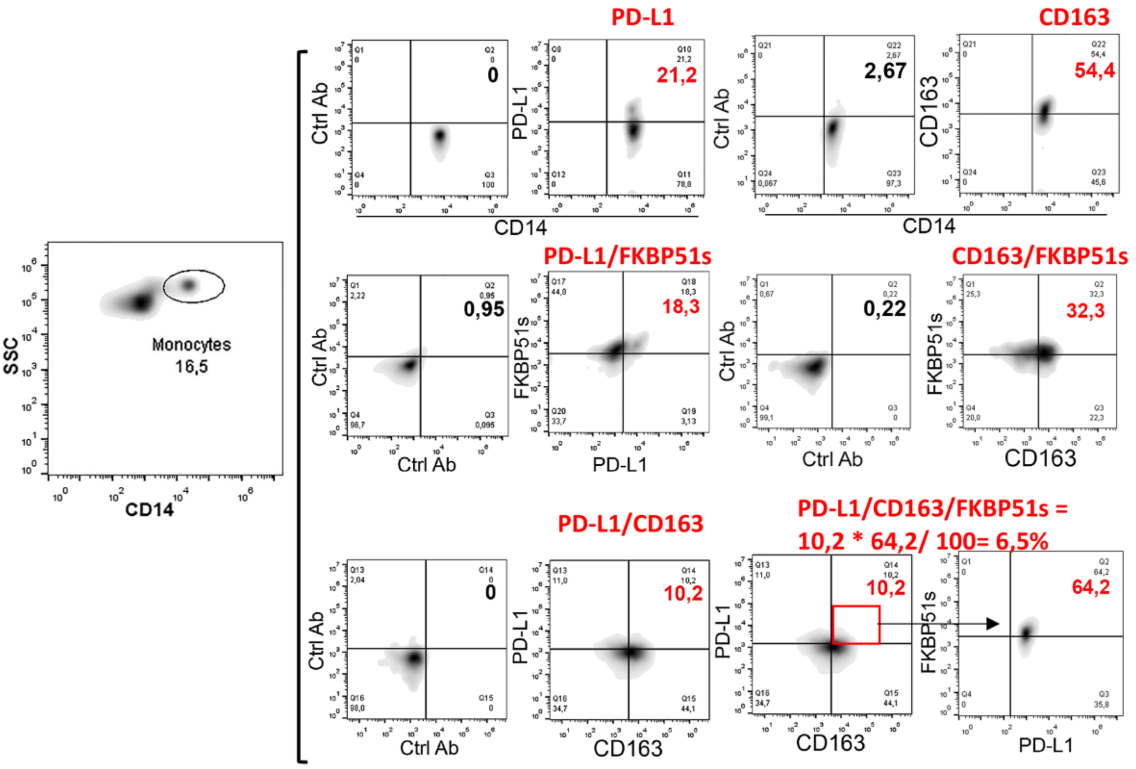

Fig. S1 Representative flow cytometry histograms of CD14/SSc-gated monocytes (left) and the CD14^{+} subsets analysed.

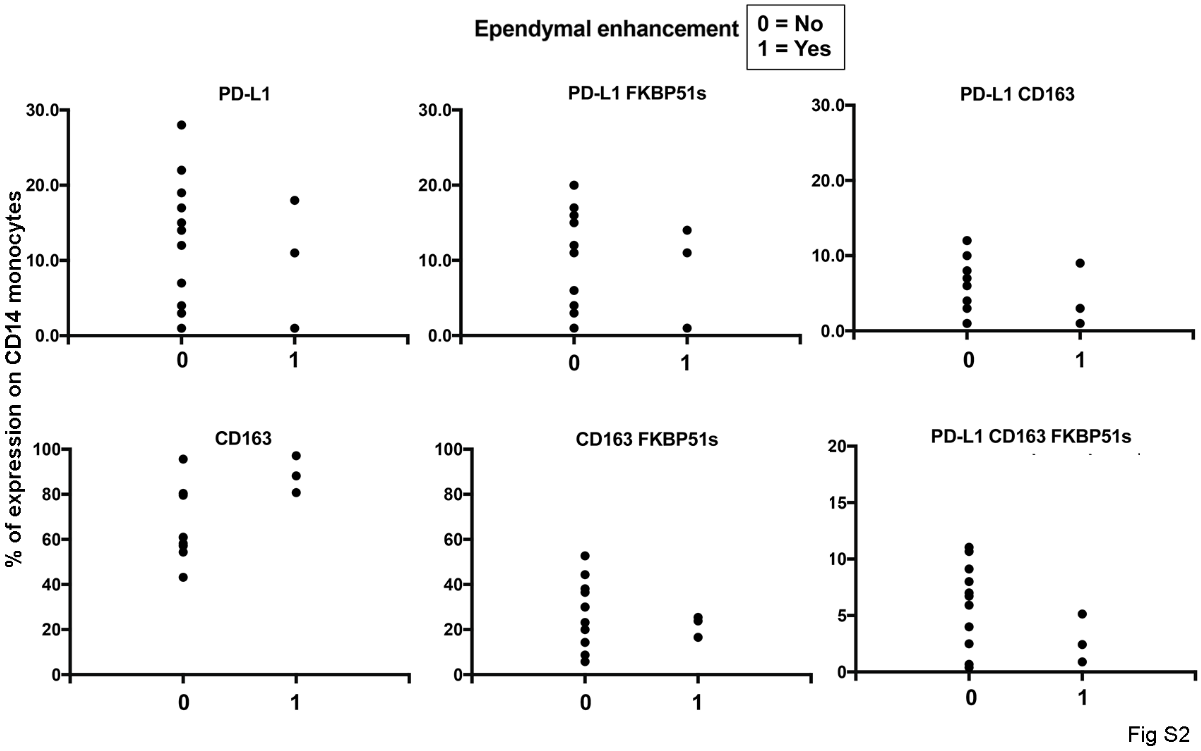

Fig. S2 M2-monocyte subsets representation according to ependymal enhancement. Graphs were generated using Prism GraphPad 7.0a Macintosh. Linear regression and Pearson correlation coefficient did not measure any linear relationship between the variables.

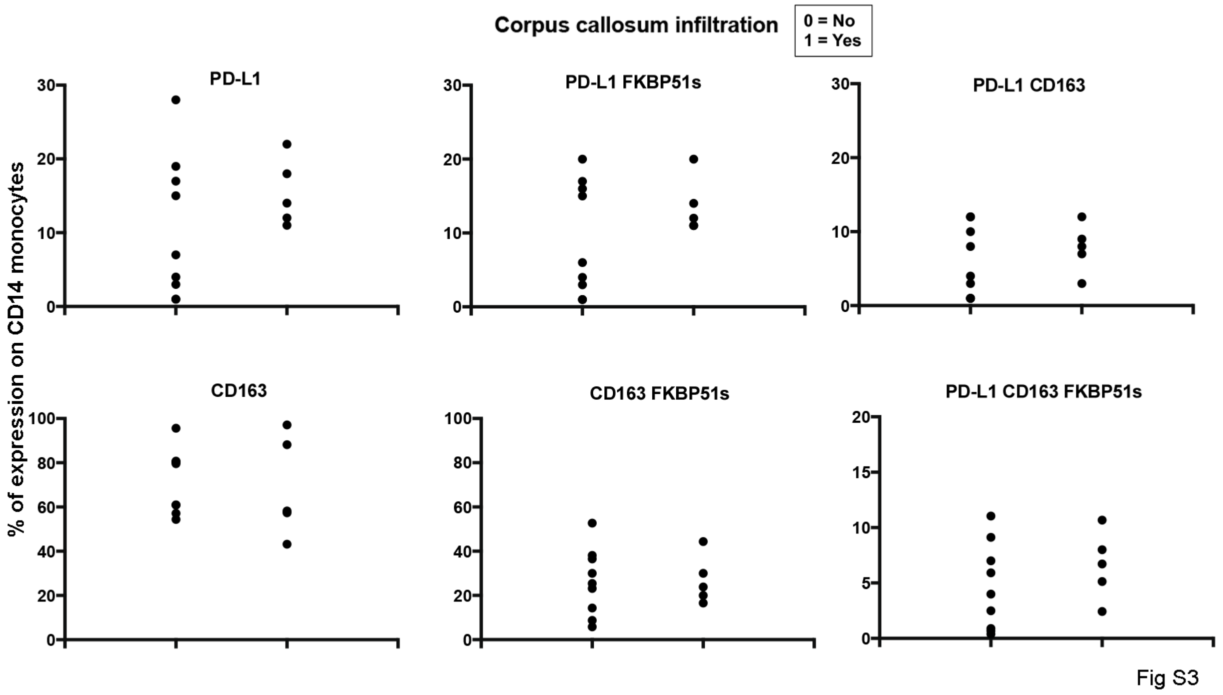

Fig. S3 M2-monocyte subsets representation according to corpus callosum infiltration. Graphs were generated using Prism GraphPad 7.0a Macintosh. Linear regression and Pearson correlation coefficient did not measure any linear relationship between the variables.

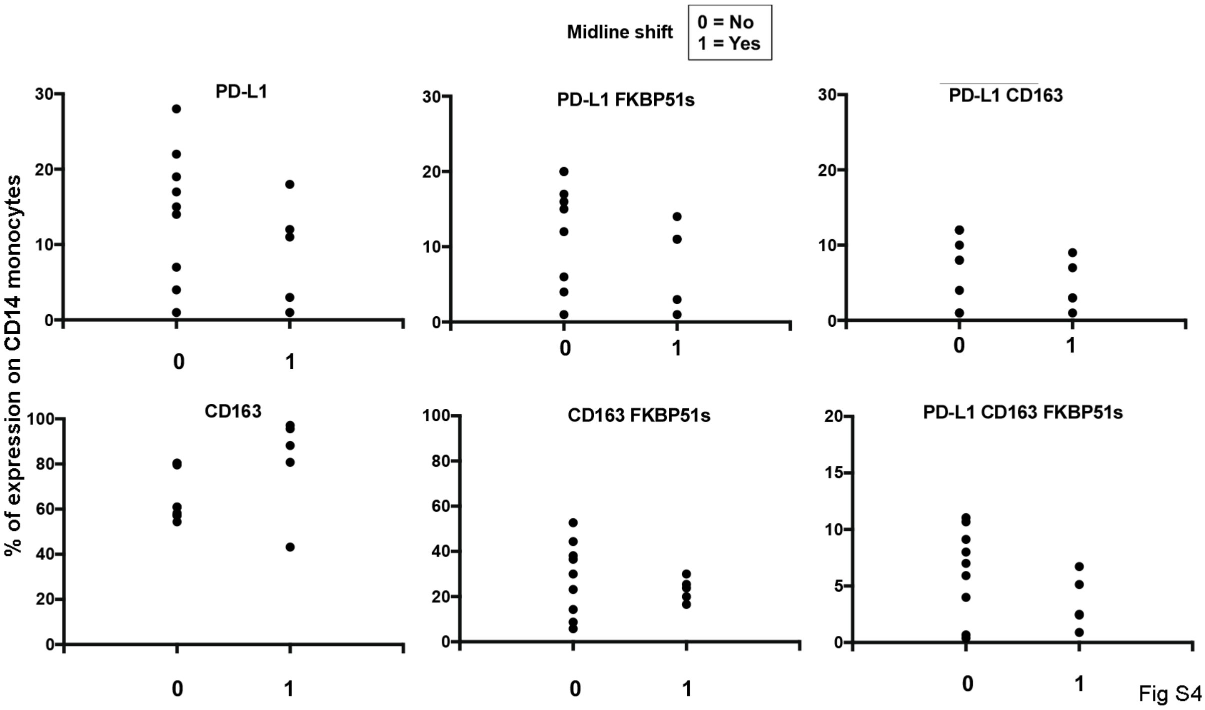

Fig. S4 M2-monocyte subsets representation according to midline shift. Graphs were generated using Prism GraphPad 7.0a Macintosh. Linear regression and Pearson correlation coefficient did not measure any linear relationship between the variables.

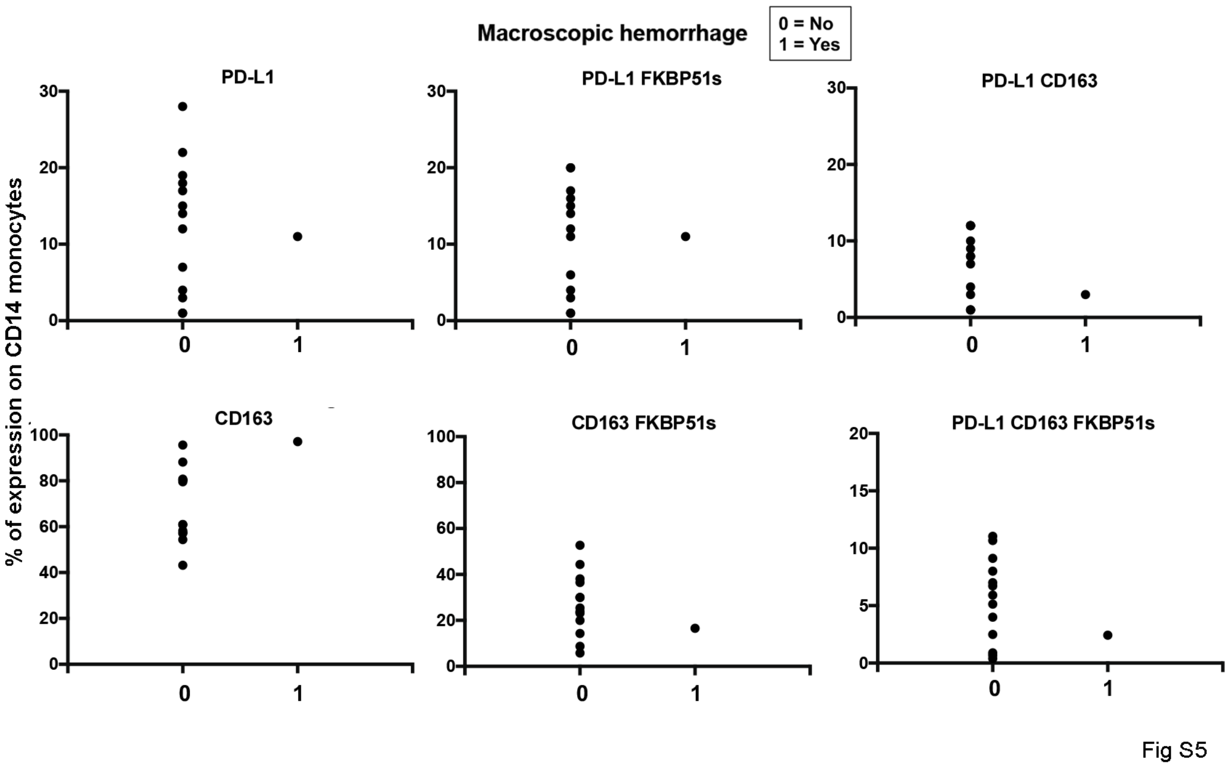

Fig. S5 M2-monocyte subsets representation according to macroscopic hemorrhage. Graphs were generated using Prism GraphPad 7.0a Macintosh. Linear regression and Pearson correlation coefficient did not measure any linear relationship between the variables.

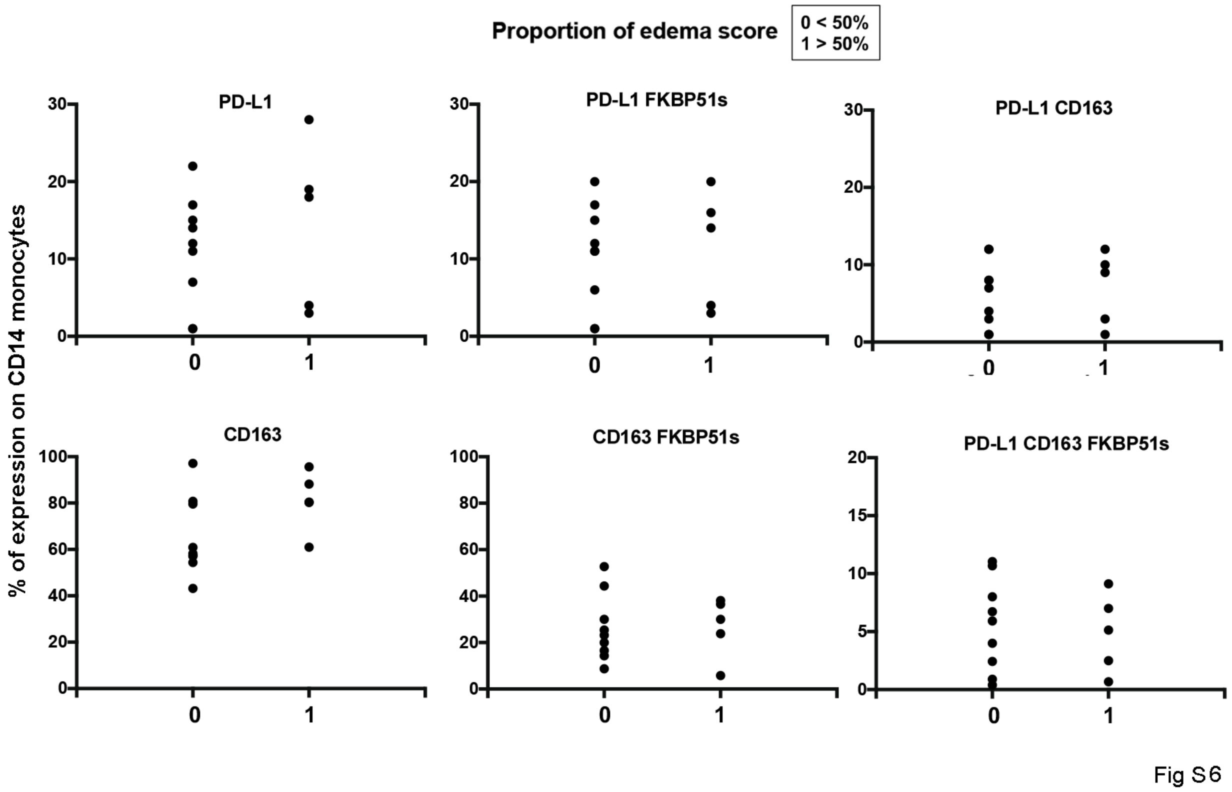

Fig. S6 M2-monocyte subsets representation according to the edema score . Graphs were generated using Prism GraphPad 7.0a Macintosh. Linear regression and Pearson correlation coefficient did not measure any linear relationship between the variables.

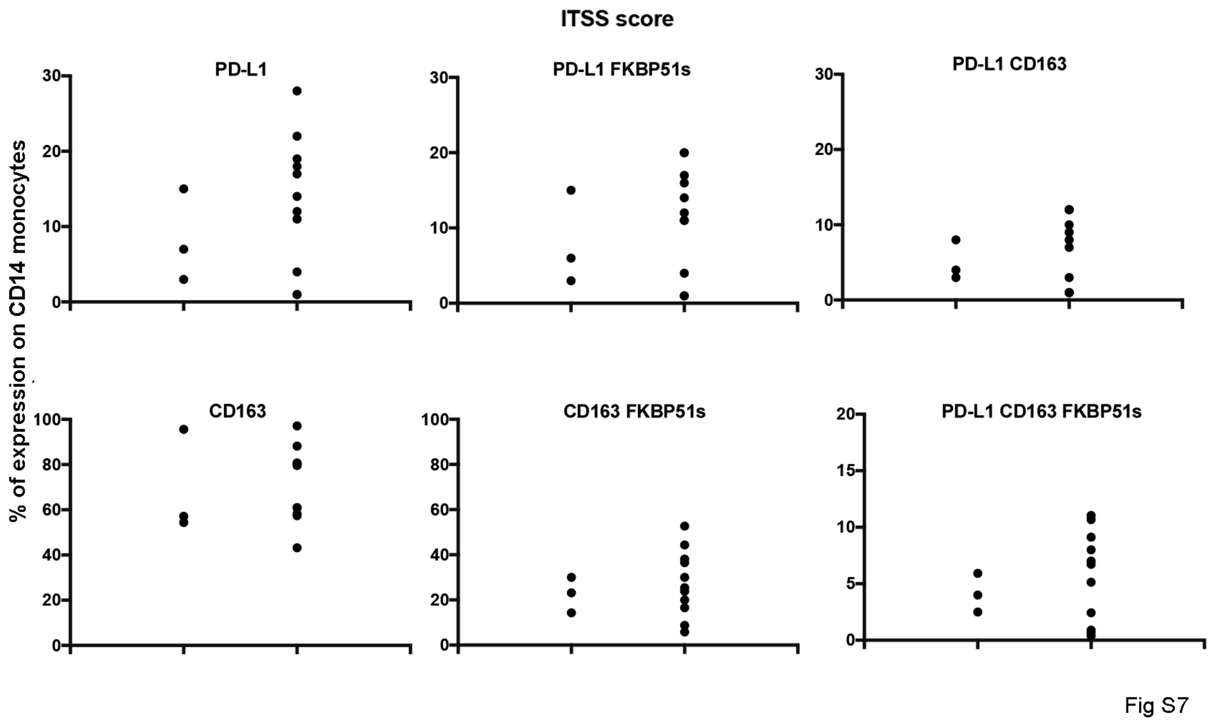

Fig. S7 M2-monocyte subsets representation according to the score of intratumoral susceptibility signal intensity (ITSS). Graphs were generated using Prism GraphPad 7.0a Macintosh. Linear regression and Pearson correlation coefficient did not measure any linear relationship between the variables.
